# Supplementary material for: The Proprioceptive System Regulates Morphologic Restoration of Fractured Bones
Source: Cell Rep. 2017 Aug 22;20(8):1775–83. doi: 10.1016/j.celrep.2017.07.073 (PMC5575358; doi:10.1016/j.celrep.2017.07.073)
Supplement: Document S1. Supplemental Experimental Procedures [file mmc1.pdf]

Cell Reports, Volume *20*

Supplemental Information

The Proprioceptive System Regulates Morphologic Restoration of Fractured Bones

Ronen Blecher, Sharon Krief, Tal Galili, Eran Assaraf, Tomer Stern, Yoram Anekstein, Gabriel Agar, and Elazar Zelzer

Supplemental Experimental Procedures

RESOURCE TABLE

REAGENT or RESOURCE	SOURCE	IDENTIFIER
Chemicals, Peptides, and Recombinant Proteins		
Clorketam (ketamine)	Vetoquinol, France	N/A
Xylazine (Sedaxylan)	Eurovet, Netherlands	23076-35-9
Buprenorphine	Vetmarket, Israel	163451
Isoflurane	AbbVie, UK	B506
Botulinum toxin A (Botox)	Allergan	N/A
Phosphate-buffered saline (PBS)	Gibco by Life Technologies, UK	14200-067
Experimental Models: Organisms/Strains		
Mouse: ICR	Envigo, Israel	Hsd:ICR (CD-1)
Mouse: C57BL/6	Envigo, Israel	C57BL/6JOlaHsd
Software and Algorithms		
R	R Development Core Team, 2016	https://www.r-project.org/
