# Supplementary material for: The Proprioceptive System Regulates Morphologic Restoration of Fractured Bones
Source: Cell Rep. 2017 Aug 22;20(8):1775–83. doi: 10.1016/j.celrep.2017.07.073 (PMC5575358; doi:10.1016/j.celrep.2017.07.073)
Supplement: Document S2. Article plus Supplemental Information [file mmc2.pdf]

Cell Reports

Report

The Proprioceptive System Regulates Morphologic Restoration of Fractured Bones

Graphical Abstract

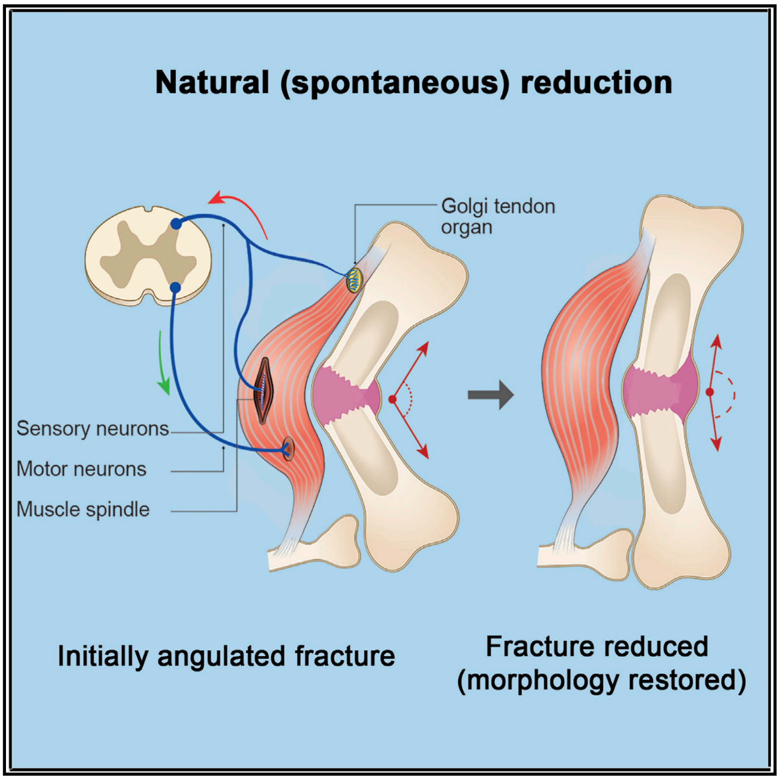

Authors

Ronen Blecher, Sharon Krief, Tal Galili, ..., Yoram Anekstein, Gabriel Agar, Elazar Zelzer

Correspondence

eli.zelzer@weizmann.ac.il

In Brief

Blecher et al. report that natural reduction, the process whereby fractured bones are realigned, fails in mutant mice lacking functional proprioceptive circuitry. Surprisingly, natural reduction was more rapid and effective in 3-month-old mice than in newborns. These findings suggest a physiological role for proprioception in non-autonomous regulation of skeletal integrity.

Highlights

- Realignment of fractured bones fails in mice lacking proprioceptive receptors
- Realignment is more rapid and effective in 3-month-old mice than in newborns
- Muscle contraction is also required for proper natural reduction
- These findings reveal a key role for proprioception in regulating fracture repair

Blecher et al., 2017, Cell Reports *20*, 1775–1783
August 22, 2017 © 2017 The Author(s).
http://dx.doi.org/10.1016/j.celrep.2017.07.073

Cell Reports
Report

OPEN ACCESS
CellPress

The Proprioceptive System Regulates Morphologic Restoration of Fractured Bones

Ronen Blecher,[1,2] Sharon Krief,[1] Tal Galili,[3] Eran Assaraf,[1,2] Tomer Stern,[1] Yoram Anekstein,[2] Gabriel Agar,[2] and Elazar Zelzer[1,4,*]
[1]Department of Molecular Genetics, Weizmann Institute of Science, Rehovot 76100, Israel
[2]Department of Orthopedic Surgery, Assaf Harofeh Medical Center, Zerrifin 70300, Israel, affiliated with the Sackler Faculty of Medicine, Tel Aviv University, Tel Aviv 69978, Israel
[3]Department of Statistics and Operations Research, Tel Aviv University, Tel Aviv 69978, Israel
[4]Lead Contact
*Correspondence: eli.zelzer@weizmann.ac.il
http://dx.doi.org/10.1016/j.celrep.2017.07.073

SUMMARY

Successful fracture repair requires restoration of bone morphology and mechanical integrity. Recent evidence shows that fractured bones of neonatal mice undergo spontaneous realignment, dubbed “natural reduction.” Here, we show that natural reduction is regulated by the proprioceptive system and improves with age. Comparison among mice of different ages revealed, surprisingly, that 3-month-old mice exhibited more rapid and effective natural reduction than newborns. Fractured bones of null mutants for transcription factor *Runx3*, lacking functional proprioceptors, failed to realign properly. Blocking *Runx3* expression in the peripheral nervous system, but not in limb mesenchyme, recapitulated the null phenotype, as did inactivation of muscles flanking the fracture site. *Egr3* knockout mice, which lack muscle spindles but not Golgi tendon organs, displayed a less severe phenotype, suggesting that both receptor types, as well as muscle contraction, are required for this regulatory mechanism. These findings uncover a physiological role for proprioception in non-autonomous regulation of skeletal integrity.

INTRODUCTION

Bone fracture repair has attracted much attention over the years. Extensive research of the cellular, molecular, and mechanical aspects of this healing process has produced an evidence-based model describing its various stages. The first step is hematoma formation around the fracture site. Next, angiogenesis commences (Ai-Aql et al., 2008; Bolander, 1992; Brighton, 1984; Cho et al., 2002; Schindeler et al., 2008), followed by formation of soft, fibrocartilaginous callus. During the subsequent stages, the soft callus undergoes chondrogenesis and then osteogenesis, producing hard, ossified callus. In the last stage, compact bone is formed and bone morphology is restored through modeling, i.e., mineral resorption at the convexity and deposition at the concavity (Einhorn, 1998; Gerstenfeld et al., 2003; Shapiro, 2008; Wilkins, 2005).

Correct morphology is vital for the function of bones (Currey, 2003; Frost, 2001; Kettelkamp et al., 1988; Ring, 2005; Weiner and Wagner, 1998). However, while extensive research has been dedicated to the final stage of bone modeling (Carpenter and Carter, 2010; Carter and Orr, 1992), the mechanisms responsible for restoring the gross shape of fractured bones immediately after the injury, and before union has been achieved, have largely been neglected. This lack of interest has, possibly, been due to the relatively successful morphological restoration that is achieved by orthopedic intervention (Christian Krettek, 2006; Connolly, 2006). Nevertheless, from an evolutionary point of view, a robust mechanism that restores bone morphology would offer a considerable advantage.

Previously, we provided direct evidence for the existence of such a mechanism. We showed that, in newborn mice, unstabilized humeral fractures undergo spontaneous realignment, which we termed ‘‘natural reduction’’ (Rot et al., 2014). Fracture realignment involved substantial movement of the two fragments, most likely driven by force generated by ossification of cartilaginous callus at the concave side of the fracture. We also showed that muscle contraction is essential for natural reduction, which failed in its absence due to premature callus ossification. The finding of this robust mechanism for fracture realignment has raised several intriguing questions. One of them is whether, as in other repair processes, the capacity for natural reduction is age restricted (Yun, 2015). Another key question relates to the mechanism that senses the location and orientation of the fracture fragments to direct realignment.

The musculoskeletal system of mammals contains a large number of proprioceptive mechanosensors. The two main types of sensors—namely, muscle spindle and Golgi tendon organ (GTO)—are found mostly throughout striated muscles and muscle-tendon junctions, respectively (Kokkorogiannis, 2004; Soukup, 1983). Via specialized afferent neuronal fibers termed ‘‘proprioceptive neurons,’’ these sensors provide an immediate feedback mechanism by forming local monosynaptic reflex arcs (Chen et al., 2003). Muscle spindles detect changes in muscle length, whereas GTOs detect changes in muscle tension (Granit, 1975; Maier, 1997; Moore, 1984). In

CrossMark

This is an open access article under the CC BY license (http://creativecommons.org/licenses/by/4.0/).

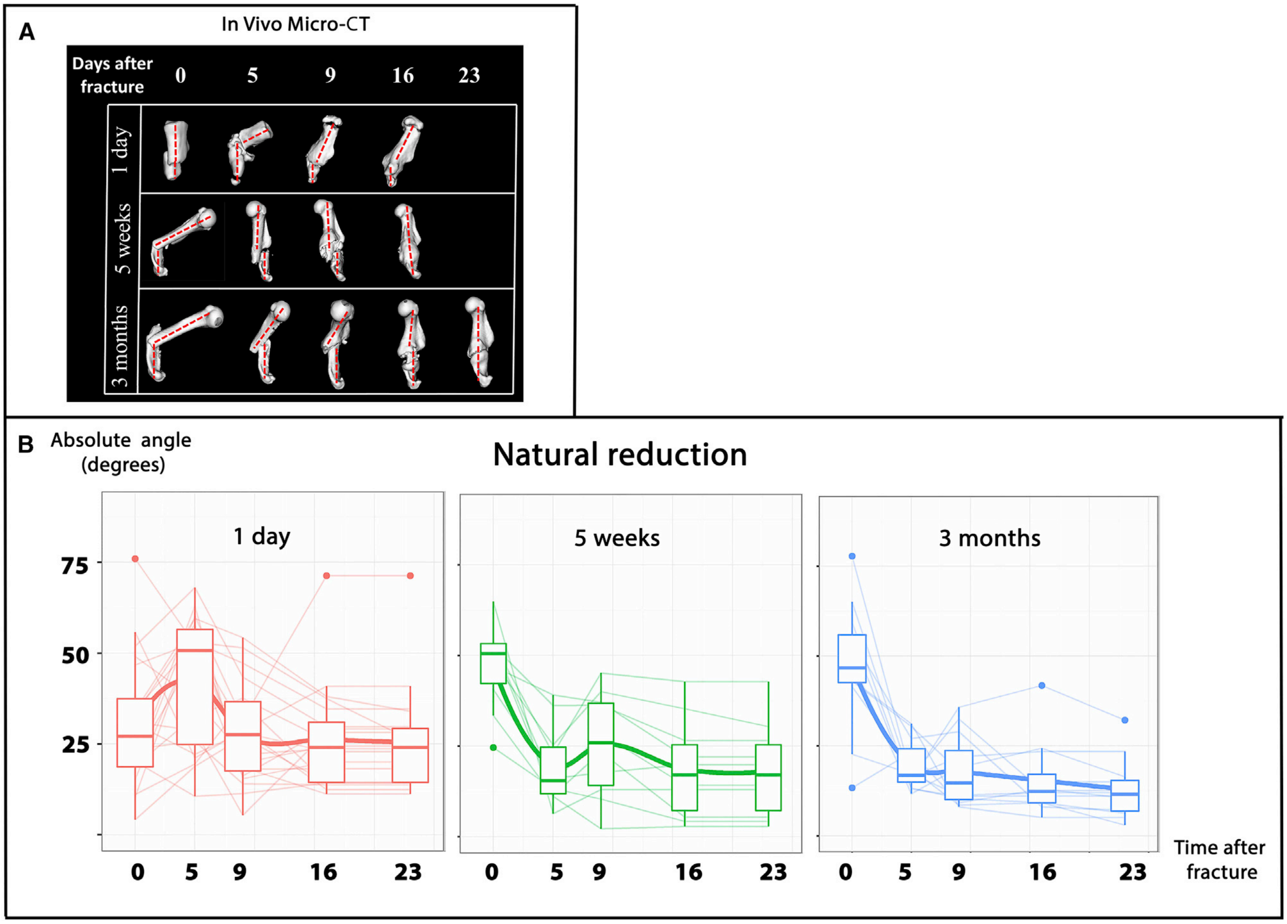

Figure 1. Age Differences in Natural Reduction

(A) In vivo CT scans of representative humeri of mice from three different age groups showing the realignment of fractured bones.

(B) Graphs summarizing natural reduction, showing the angle between fracture fragments during healing in three age groups. Thin lines connect readings of individual fractures, a continuous thick line indicates the mean, thick lines within boxes indicate the medians, box limits indicate the 25th and 75th percentiles, and whiskers extend to the 5th and 95th percentiles. All age groups exhibited a capacity for natural reduction. From P1 to P90, older animals exhibited faster and a more robust realignment. Newborn mice displayed a reversal in the healing pattern observed in other age groups, which was significant at the post-fracture intervals days 0–5 ($p = 0.0000013$) and days 5–9 ($p = 0.004$). At day 23, the fractures induced in 3-month-old mice displayed less deformity relative to those induced in newborns ($p = 0.01$).

recent years, several key factors regulating the formation of these arcs have been identified. For example, proper connectivity of both sensor types was found to depend on the expression of Runt-related transcription factor 3 (*Runx3*) in proprioceptive neurons in the dorsal root ganglia (DRG) (Inoue et al., 2003; Levanon et al., 2002). *Egr3*, a member of the zinc finger family of transcription factors, was shown to regulate the formation of muscle spindles, specifically (Oliveira Fernandes and Tourtellotte, 2015; Tourtellotte and Milbrandt, 1998).

In this work, we propose that the neuronal system and, in particular, the proprioceptive system act as a super-mechanism orchestrating natural reduction. We show that natural reduction is more effective in skeletally mature mice, as compared to newborns, and, finally, that muscle spindles and GTOs act additively to regulate this process.

RESULTS

Natural Reduction Improves over the First Postnatal Months

Previously, we demonstrated that humeral fractures induced in neonatal mice can undergo spontaneous morphological restoration, which we termed "natural reduction" (Rot et al., 2014). An interesting question that has remained open is whether, as in other repair processes (Gould et al., 2015; Sanz et al., 1999), natural reduction is age restricted. In order to address this question, we performed humeral fractures in mice of three age groups: 1 day old, 5 weeks old, and 3 months old. To study the process of natural reduction, mice were scanned by in vivo computed tomography (CT). In our previous work, in order to focus on the role of fracture callus, bones were first scanned 5 days after fracture induction (Rot et al., 2014). In the present study, in order to obtain

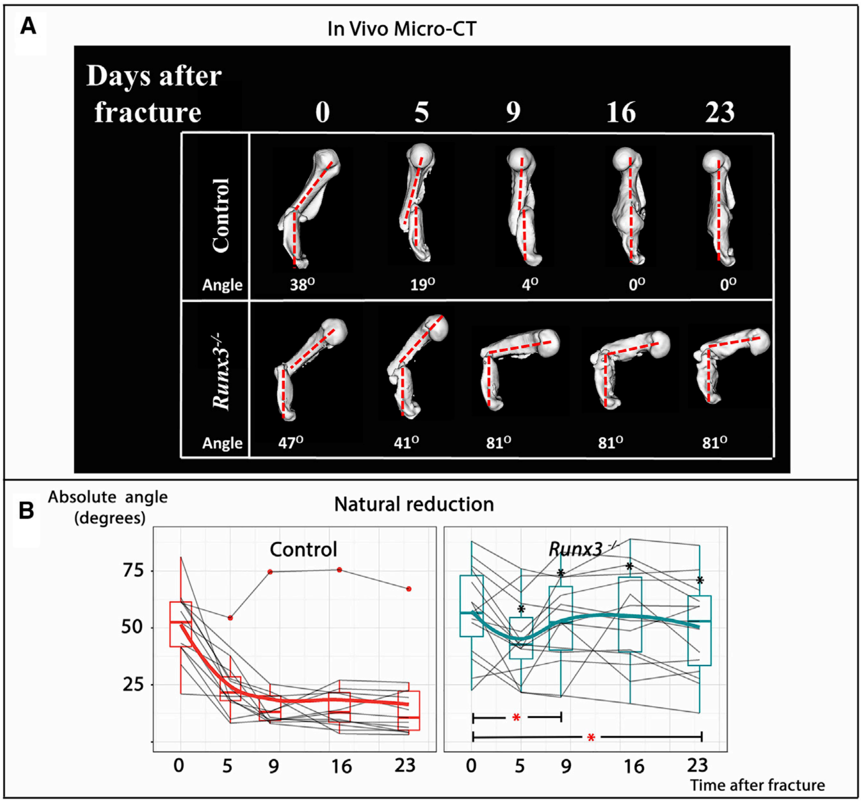

Figure 2. Failed Natural Reduction in *Runx3*$^{-/-}$ Mice

(A) Natural reduction in sequential in vivo CT scans of representative mutant *Runx3*$^{-/-}$ mice and control littermates.

(B) Graphs summarizing natural reduction in both groups. Natural reduction completely failed in the majority of *Runx3*-deficient mice, where an initial improvement in angulation was not maintained, resulting in significantly increased angulation from PF5 onward. Reduction rate was also significantly reduced during both the PF0–PF9 and PF0–PF23 intervals. Black asterisks indicate significant differences ($p < 0.05$) in mean angulation between mutant and control mice at specific time points, whereas red asterisks indicate significant differences during specific time intervals.

a complete picture of the dynamics of the process, we repeatedly scanned mice from the induction of fracture (day 0) until a continuous bony tissue bridging the two fragments was evident (days 16–23). As expected, fractured bones underwent natural reduction (Figure 1). However, comparison of the course of reduction in the three age groups revealed surprising differences. When fractures were induced in mice at post-natal day (P)1, in most cases, the angulation increased by day 5 and then decreased by days 9–16. In sharp contrast, in the older mice, a dramatic decrease in angulation, i.e., the abnormal angle formed between the fracture fragments, was observed already at day 5. In general, the oldest mice, 3 months of age, exhibited the fastest and most robust reduction and, on average, the least deformity in the final scan.

Together, these results indicate that, in the first months after birth, the capacity for morphological restoration of long bones may improve with age rather than being limited by it. In addition, our finding that, unlike in newborns, extensive reduction in older mice is already achieved in the first few days, before substantial formation of cartilaginous callus (Vortkamp et al., 1998), infers the involvement of a mechanism unrelated to fracture callus in the induction of realignment.

Natural Reduction Is Severely Impaired in *Runx3*-Deficient Mice

In search of a mechanism that would induce a robust and immediate reaction in fractured bones, we considered the involvement of a neural system. Proprioceptive mechanosensors, which respond to biomechanical changes by adapting local muscle tension, may serve in the control of natural reduction by sensing the position of the two fracture fragments to direct realignment. To study the hypothesis that muscle spindles and GTOs are involved in the regulation of natural reduction, we used *Runx3* transcription factor as a molecular entry point. *Runx3* is necessary for the survival of *TrkC*-positive proprioceptive neurons, whose absence results in the inactivation of muscle mechanosensors (Inoue et al., 2003; Levanon et al., 2002). Thus, we performed humeral fractures in 3-month-old *Runx3* knockout (KO) and control wild-type (WT) mice, leaving all fractures unstabilized, and followed the course of fracture repair using in vivo CT scans. As seen in Figure 2, natural reduction completely failed in *Runx3* mutant mice, resulting in significantly increased angulations at post-fracture day (PF)23.

Runx3 Expression in Limb Mesenchyme Is Dispensable for Natural Reduction

Our results clearly demonstrate the centrality of *Runx3* in the process of natural reduction. RUNX3 was previously shown to regulate numerous processes in different tissues, including osteogenic (Bauer et al., 2015; Lian et al., 2004) and chondrogenic (Stricker et al., 2002; Yoshida et al., 2004) cell populations. Therefore, to eliminate the possibility that the role of *Runx3* in natural reduction is proprioception independent, we deleted its expression in limb mesenchyme-derived cell populations—namely, osteoblasts and chondroblasts—using the *Prx1-Cre* mouse as a deleter (Logan et al., 2002). Mutant mice in which *Runx3* was deleted from all mesenchymal lineages (*Prx1-Cre-Runx3*$^{f/f}$) underwent the same fracture induction procedure as described earlier. As seen in Figure 3A, similar radiographic follow-up showed that the specific ablation of *Runx3* from both chondrocytes and osteoblasts did not affect natural reduction, indicating that mesenchymal *Runx3* expression is dispensable for this process.

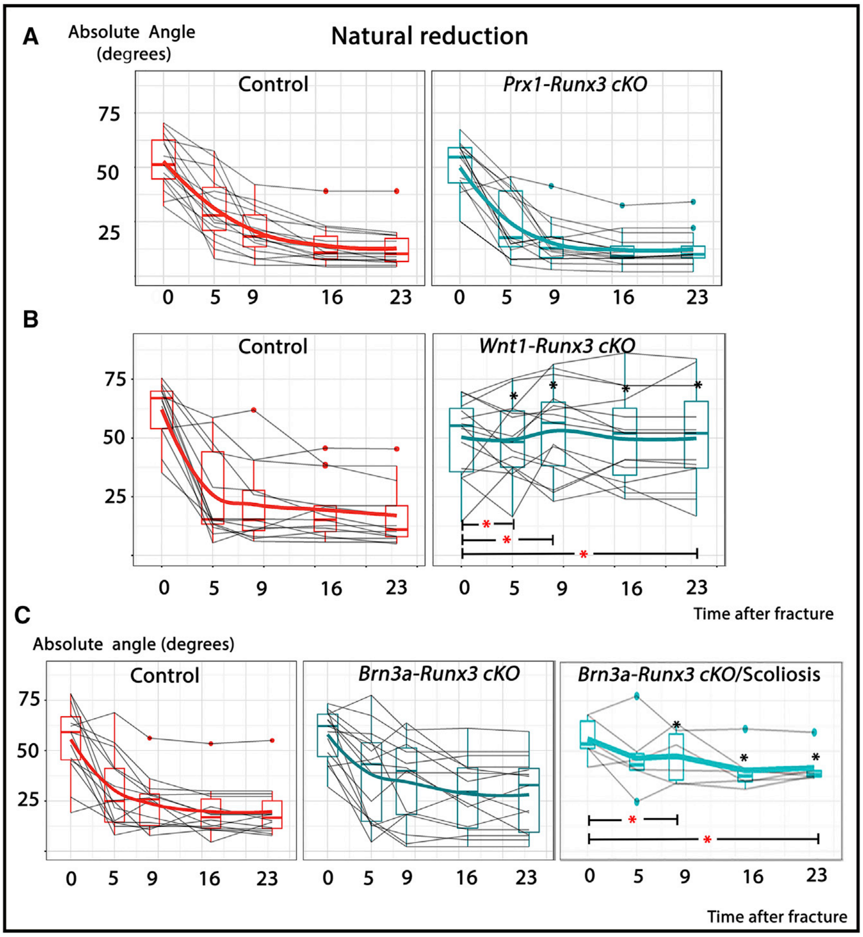

Figure 3. Failed Natural Reduction in Neural but Not Mesenchymal *Runx3* Conditional Knockout Strains

(A–C) Graphs summarizing natural reduction in *Prx1-Runx3* (mesenchyme) (A), *Brn3a-Runx3* (sensory peripheral) (B), and *Wnt1-Runx3* (pan-peripheral) (C) cKO mice show substantial impairments in both neural cKO strains. (A) Natural reduction was not affected in mesenchymal *Runx3* cKO. (B) In the *Brn3a*-driven cKO, impaired natural reduction was evident but did not reach significance level. Analysis of *Brn3a-Runx3* cKO mice with spinal deformity (on the right) showed increased mean angulations measured at PF9, PF16, and PF23, relative to the control. The rate of reduction significantly decreased at the intervals PF0–PF9 and PF0–PF23. (C) Complete neuronal ablation of *Runx3* in the *Wnt1*-driven cKO strain impaired natural reduction to a larger extent. Relative to control mice, mean angulation increased significantly at every measured time point from PF5 thereafter, resulting in substantially larger mean final angulation (49.9° as compared to 19°), and reduction rates were slower at intervals PF0–PF5, PF0–PF9, and PF0–PF23. Black asterisks indicate significant differences ($p < 0.05$) in mean angulation between mutant and control mice at specific time points, whereas red asterisks indicate significant differences during specific time intervals.

Specific Neuronal Ablation of *Runx3* Impairs Natural Reduction

Having excluded a possible effect of *Runx3* expression in osteoblasts or chondrocytes on natural reduction, we proceeded to examine the effect of specific neural ablation. For that, we used two neural Cre drivers. *Brn3a* is expressed by primary sensory neurons of the cranial nerves and in the DRG (Dykes et al., 2011; O'Donovan et al., 2014), whereas *Wnt1-Cre* acts in the entire peripheral nervous system (Brault et al., 2001). Recently, it was reported that *Brn3a-CreER*-mediated conditional knockout (cKO) of *Runx3* (*Brn3a*-CreER-*Runx3*$^{floxed/null}$) resulted in partial ablation of *Runx3*-positive cells, whereas using a *Wnt1-Cre* driver (*Wnt1-Cre-Runx3*$^{floxed/floxed}$) caused a complete ablation of *Runx3* expression from the DRG (Blecher et al., 2017). Indeed, functional evaluation revealed that, whereas the gait pattern of mature *Brn3a-Runx3* cKO mice was only partially affected, *Wnt1-Runx3* cKO animals displayed severely uncoordinated gait, similarly to the phenotype seen in *Runx3* knockout (KO) mice (Blecher et al., 2017).

To examine the direct effect of neuronal *Runx3* loss of function on natural reduction, we performed fractures in 3-month-old cKO mice and control littermates. As shown in Figure 3B, blocking *Runx3* expression, using *Wnt1-Cre* as a deleter, resulted in complete failure in natural reduction, as in *Runx3* mutants. In comparison, using the *Brn3a* deleter led to a mixed phenotype; i.e., in some mice, natural reduction failed, whereas in others, it appeared to proceed normally (Figure 3C). The variation we observed in natural reduction most likely resulted from the partial ablation of *Runx3* in the DRG by the *Brn3a-CreER.* To validate this supposition, we assessed the efficiency of monosynaptic reflex perturbation independently, using the spinal deformity of *Brn3a-Runx3* cKO mice. Recently, we demonstrated that disruption of the proprioceptive system led to acquired spinal deformity (scoliosis) (Blecher et al., 2017). Indeed, mice that exhibited scoliosis also displayed failed natural reduction, as compared to control littermates (Figure 3C).

Collectively, these results clearly demonstrate that *Runx3* function, specifically in neural tissues, is essential for natural reduction. This finding further supports the notion that natural reduction is regulated neuronally and, in particular, by proprioceptive mechanosensors.

Natural Reduction Is Dependent on Both Types of Sensors

As mentioned earlier, the two main types of proprioceptive mechanosensors are the muscle spindle and the GTO (Granit, 1975; Maier, 1997; Moore, 1984). To validate the role of the proprioceptive system in the regulation of natural reduction and determine the relative contributions of the two sensor types, we performed fractures in mature (P90) mice deficient in *Egr3*, in whom muscle spindles fail to survive but GTOs are retained

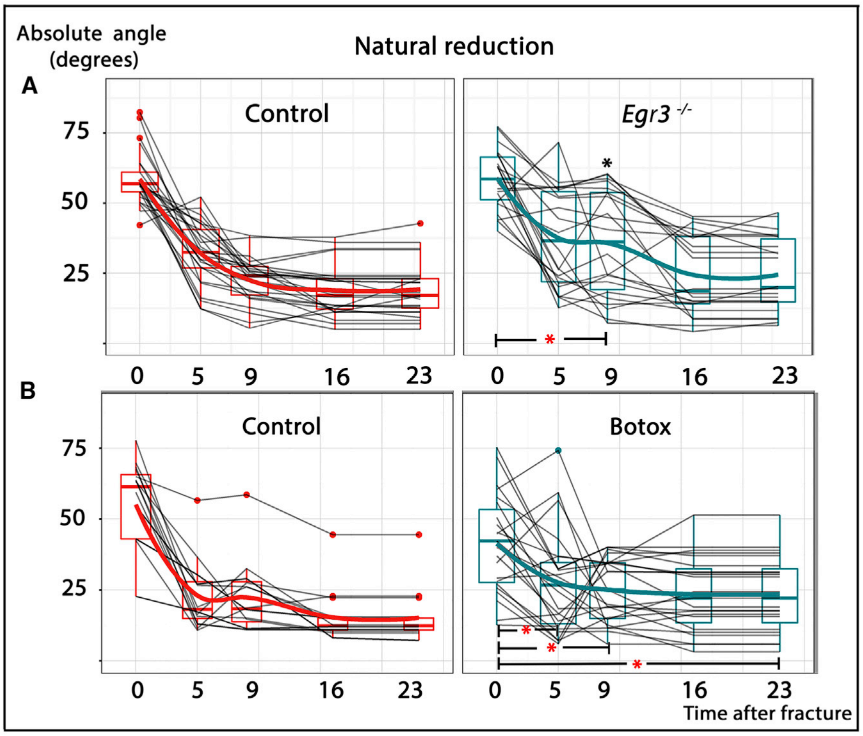

Figure 4. Impaired Natural Reduction upon Muscle Spindle Ablation and Local Muscle Inactivation

(A and B) Graphs summarizing natural reduction in muscle-spindle-deficient animals (*Egr3* KO) (A) and upon local muscle inactivation (Botox) (B). In spite of seemingly normal initiation, natural reduction progressed less rapidly and less efficiently in *Egr3* KO mice lacking muscle spindles, which displayed a significantly reduced rate of reduction at PF9 compared to PF0 and increased mean angulation at PF9, relative to control mice. Partial paralysis, induced by injecting Botox prior to fracture induction, resulted in significantly decreased rates of reduction at intervals PF0–PF5, PF0–PF9, and PF0–PF23. Black asterisks indicate significant differences ($p < 0.05$) in mean angulation between mutant and control mice at specific time points, whereas red asterisks indicate significant differences during specific time intervals.

(Chen et al., 2002). As seen in Figure 4A, the specific ablation of muscle spindles interfered with the normal sequence of natural reduction. This finding reinforces the involvement of muscle proprioceptors in the regulation of natural reduction. However, comparison between the outcomes of the perturbation either of muscle spindles alone or of muscle spindles and GTOs clearly showed that both types are needed to exert the full effect of natural reduction.

Natural Reduction Is Dependent on Muscle Activity

The involvement of proprioceptive mechanosensors in natural reduction suggested that their effect on bone realignment is mediated by controlling muscle activity. To test this postulate, we induced partial paralysis of the muscles adjacent to the fracture site by injecting botulinum toxin (Botox) locally. Repeating injections starting prior to fracture induction allowed paralysis to develop before fractures were induced. The total amount injected was limited to induce partial paralysis but avoid systemic toxicity. Fracture induction and radiographic assessment of realignment were performed as described earlier. As seen in Figure 4B, natural reduction was significantly impaired in partially paralyzed WT mice, resulting in increased final angulation. In addition, reduction rate was significantly slower at intervals PF0–PF5, PF0–PF9, and PF0–PF23. These results support the notion that muscle activity is necessary for the ability of the proprioceptive system to regulate natural reduction.

DISCUSSION

The ability to restore skeletal morphology after a traumatic insult to bone integrity has granted vertebrates a considerable evolutionary advantage. In spite of extensive clinical research into the association between fracture realignment and functional outcome (Ellsasser et al., 1975; Fogel and Morrey, 1987; Lam, 1964), little has been noted on the realignment process itself (Husain et al., 2008; Schultz, 1939, 1944). Recently, we have described the process of natural reduction, whereby bone morphology is restored in neonatal mice (Rot et al., 2014). However, the questions of whether natural reduction is age restricted and regarding the mechanism that regulates it have remained open. Here, we show that, surprisingly, natural reduction is more efficient in mature mice; that proprioceptive mechanosensors control and guide fracture realignment; and, finally, that both main sensors, muscle spindles and GTOs, probably act additively to orchestrate natural reduction.

The long-known mechanosensing capability of bones has, so far, been considered autonomous (Currey, 2002, 2003; Burr and Allen, 2014; Frost, 2001; Weiner and Wagner, 1998). Bones react to changes in mechanical loading by adapting their morphology (Robling et al., 2006; Sharir et al., 2011), mineral composition, and density (Bach-Gansmo et al., 2016; Ellman et al., 2013) to cope with the new mechanical requirements. At the cellular level, it has been reported that chondrocytes (Lee et al., 2000; Wann et al., 2012), osteoblasts (Davidson et al., 1990), and osteocytes (Huiskes et al., 2000) all sense and respond to mechanical stimulation. Similarly, during fracture repair, callus has been shown both experimentally (Probst and Spiegel, 1997; Thompson et al., 2002) and clinically (Aro and Chao, 1993) to maintain these sensing capabilities, a principle that is widely utilized in the clinical practice to guide therapeutic decisions (Aro and Chao, 1993; Christian Krettek, 2006; Ilizarov, 1990). Our finding of a proprioception-mediated mechanism that monitors and restores bone integrity adds another level of non-autonomous regulation to the current view of mechanosensing during fracture healing.

Musculoskeletal development and growth are regulated by cross-interactions among different tissues (Blitz et al., 2009;

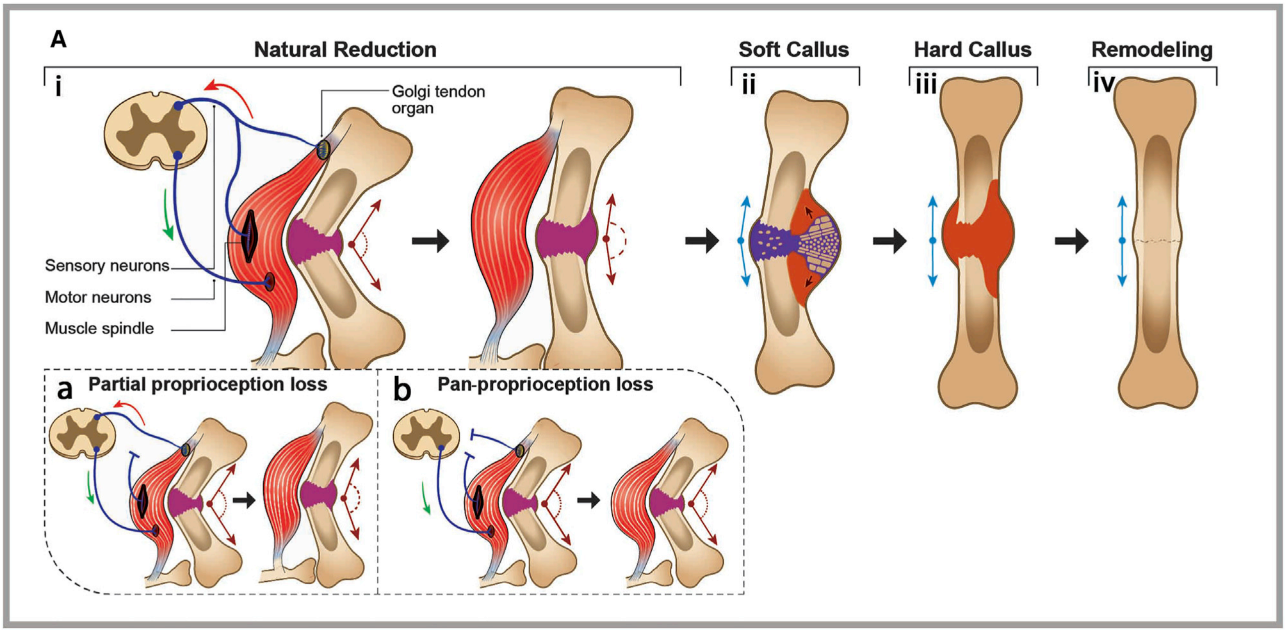

Figure 5. Fracture Healing Is Initially Guided by the Proprioceptive System

In the suggested revised model of fracture repair (A), both muscle mechanosensor types act additively to guide natural reduction (i). Later, asymmetrical callus growth (ii) further drives bone alignment, providing optimized conditions for the later stages of callus ossification (iii) and remodeling (iv).
Partial (a) or complete (b) loss of proprioception results in corresponding levels of impairment in natural reduction.

Kahn et al., 2009; Shwartz et al., 2012; Zelzer et al., 2014). For example, skeletal muscles have been shown to regulate the morphology and mineral content of the developing bone (Sharir et al., 2011). A role for cross-tissue interactions also in fracture repair was indicated in a recent report suggesting that muscle-derived satellite cells actively participate in the process by expressing various growth factors (Abou-Khalil et al., 2015). Our findings that fracture repair is regulated by the monosynaptic stretch reflex circuitry and dependent on muscle activity further underscores the importance of these interactions between musculoskeletal tissues.

Proprioceptive mechanosensors provide constant homeostatic regulation of skeletal muscle tension to prevent potentially injurious over-activation (Windhorst, 2007). However, in recent years, it has become increasingly clear that these mechanosensors are involved in additional processes, such as the reported role of muscle spindles in directing locomotor recovery following spinal cord injury (Takeoka et al., 2014). Recently, we showed that, in the absence of an intact proprioceptive circuitry, peripubertal mice develop a spinal deformity without vertebral dysplasia, similar to the human condition known as adolescent idiopathic scoliosis (Blecher et al., 2017). In this study, we uncover another example of proprioceptive regulation of the skeleton; namely, in fracture repair. The accumulating data on the involvement of the proprioceptive system in both maintenance and repair of various systems and tissue types increase substantially the scope of known physiological functions of this system.

Interestingly, in both spine alignment and fracture healing, the proprioceptive system is key in the restoration of disrupted skeletal equilibrium. This common feature implicates this system as a central regulator of the response to pathological processes affecting the musculoskeleton. However, whereas in the spine, proprioceptors seem to act continuously as gatekeepers that prevent pathology from occurring, in a fractured bone, they provide an emergency response to actively restore the lost balance.

Several pieces of evidence support the existence of a robust mechanism of natural reduction that rapidly restores bone morphology following injury. Humeral birth fractures with severe angulations commonly heal well without intervention and with little residual deformity (Husain et al., 2008). Additionally, studies of primate skeletons have documented high rates of well-healed fractures, which were also marked by minimal residual deformity, further indicating that morphological restoration can occur spontaneously (Schultz, 1939, 1944). Our finding that natural reduction is regulated by the proprioceptive system provides the mechanism underlying this repair process.

Interestingly, and in contrast to the common knowledge on repair processes, we show that the potential for natural reduction is not age restricted but, rather, improves with age. A possible mechanistic explanation for this reverse trend is that, although muscle spindles are present at birth in mice, their sensory endings continue to develop as late as 30–40 days postnatally (Maeda et al., 1985; Osawa et al., 1988). Similarly, functional studies in humans have shown that the ability to perform proprioception-specific tasks increases from childhood into adolescence (Goble et al., 2005; Pickett and Konczak, 2009), further strengthening the notion that proprioceptive efficiency is positively correlated with both increased age and improved natural reduction. Given the probability to sustain such an injury, which

was reported to be higher at mature age in gibbons (Schultz, 1944), and its presumed functional impact, the difference in the capacity to restore morphology between bone and other organs highlights the evolutionary importance of this unique program.

Traditionally, the research of fracture repair has focused on the stages of inflammation, angiogenesis, chondrogenesis, and remodeling, overlooking the immediate reaction to the injury. Possible explanations for this exclusion are the long-standing practice of manually reducing displaced fractures and the introduction of modern orthopedics and anesthesia, which allow for surgical correction (Christian Krettek, 2006). Based on our findings, we propose a revision in the conventional model of fracture repair (Figure 5). We suggest proprioception as the mechanism that both senses the position of the fracture fragments and guides natural reduction via reflex-mediated muscle activity. According to this model, asymmetric muscle activation triggered by proprioceptive mechanosensors may promote rapid and effective fracture realignment. Moreover, we argue that this mechanism is activated at the earliest stage of fracture repair and before the previously found asymmetrical callus growth and, thereby, substantially optimizes the healing process and its outcome.

The present study has several limitations. First, although we present a model in which various proprioceptive-specific perturbations result in impaired fracture realignment, direct evidence for the actual realignment process at the sensor level is lacking. Second, based on the phenotype observed in the *Wnt1-Runx3* cKO mice, we concluded that natural reduction is a neuronal event. However, given the recent reports of *Wnt1* expression in bone (Glejzer et al., 2011; Isern et al., 2014), we cannot exclude the possibility that the observed phenotype resulted from *Runx3* ablation from both osseous and neuronal tissues. Finally, the various proprioception-deficient strains analyzed also displayed varying degrees of ataxia. Fracture healing has long been considered to be influenced by mechanical factors, such as weight bearing. Thus, gait impairment could play a causative role in the observed phenotype, a possibility that could not be excluded by the tools we used in this study.

In summary, we show here that proprioceptive mechanosensors regulate the realignment of fractured bones, an effect that is mediated by muscle activity. These findings shed light on the non-traditional roles of the proprioceptive system in skeletal regulation. The identification of additional molecular players that participate in the transduction of the mechanical signals to the fracture site will further elucidate this regulatory network. Finally, growing evidence for multiple roles of proprioceptors in the musculoskeletal system calls for a major revision of the function of the proprioceptive system, which could reveal more yet-unknown interactions with potential medical implications.

EXPERIMENTAL PROCEDURES

Further details and an outline of resources used in this work can be found in Supplemental Experimental Procedures.

Animals

All experiments involving mice were approved by the Institutional Animal Care and Use Committee (IACUC) of the Weizmann Institute. The generation of *Runx3* null (KO; ICR background) (Levanon et al., 2002), *Egr3* null (KO; C57BL/6 background) (Tourtellotte and Milbrandt, 1998), LoxP-flanked (floxed) *Runx3* (*Runx3$^{LoxP/LoxP}$*) (Levanon and Groner, 2009), *Prx1-Cre* (Logan et al., 2002), *Brn3a-CreER^{T2}* (O'Donovan et al., 2014), and *Wnt1-Cre* (Danielian et al., 1998) mice has been described previously. *Runx3$^{f/f}$*/*Prx1-Cre*, *Runx3$^{f/f}$*/*Wnt1-Cre*, and *Runx3$^{f/null}$*/*Brn3a-CreER^{T2}* mice were generated by crossing *Runx3$^{LoxP/LoxP}$* mice to corresponding Cre strains. In all timed pregnancies, the plug date was defined as embryonic day (E)0.5.

Fracture Induction

Anesthesia was induced by intraperitoneal injection of ketamine (35 mg/kg) and xylazine (4.5 mg/kg), with a 27G needle. After sterile preparation of the arm skin, a small incision was made anteriorly above the elbow. The anterior arm fascia was exposed, and blunt dissection enabled a direct approach to the humerus. Using a curved blade, the bone was sawn 2–3 mm below the deltoid tuberosity. In order to decrease variance in fracture locations, each fracture was induced only after direct visualization of the tuberosity's tip. After a complete fracture was verified, the wound was closed using 5-0 nylon suture (Ethicon, Somerville, NJ). Following the procedure, mice were allowed to recover on a heated pad and then were placed in recovery cages. Pain was managed using buprenorphine (0.05–0.1 mg/kg, administered subcutaneously postoperatively and 8 hr following the procedure).

In Vivo Micro-CT

Prior to micro-CT scanning, mice were anesthetized with isoflurane (2-chloro-2-(difluoromethoxy)-1,1,1-trifluoro-ethane). Scans were performed with a TomoScope 30S Duo scanner (CT Imaging) equipped with two source-detector systems. The operation voltage of both tubes was 40 kV, integration time was 90 ms, and the isotropic resolution was 76 mm. Data were analyzed using MicroView software (GE Healthcare, v.5.2.2).

Measurements of Fracture Angulation

To measure the angle between fracture fragments, the isosurface was first extracted to generate a 3D representation of the ossified bone. Next, the formed surface was manually repositioned and rotated to align the sagittal and coronal planes of the bone with the (x,z) and (y,z) planes of the image grid, respectively. Then, the angle between proximal and distal fragments was measured manually in each plane, and the fracture angle was calculated using a mathematical equation, as described previously (Rot et al., 2014).

Muscle Paralysis by Botox

Mice were anesthetized as described previously. After sterile preparation of the arm skin, two small incisions were performed in the anterior and posterior aspects of the arm to approach the biceps and triceps muscles, respectively. Next, botulinum toxin A (Botox, Allergan; 0.15 U, 10 μl final volume) was separately injected into each muscle. The procedure was performed 1 week prior to fracture induction and was repeated at 1-week intervals until osseous bridging was noted in CT scans. Control mice were given intramuscular injections of PBS by the same procedure.

Statistical Analysis

Repeated measurements of fracture angles were analyzed for the mean, median, SD, and Pearson's moment coefficient of skewness. Comparison between groups was done using a Wilcoxon rank-sum test, which does not require assuming normal distribution of data. To control the false discovery rate (FDR), p values were adjusted for multiple comparisons using the Benjamini-Hochberg procedure. Both adjusted and unadjusted p values are below the 0.05 threshold, and only the adjusted p values are presented in the text. Calculations were made using R software (R Development Core Team, 2016).

SUPPLEMENTAL INFORMATION

Supplemental Information includes Supplemental Experimental Procedures and can be found with this article online at http://dx.doi.org/10.1016/j.celrep.2017.07.073.

AUTHOR CONTRIBUTIONS

R.B. designed and conducted the experiments, analyzed the data, and wrote the paper. S.K. conducted the experiments. T.G. conducted statistical analyses. E.A. conducted the experiments. T.S. analyzed the imaging data. Y.A. and G.A. interpreted the results and provided comparative clinical and anatomical advice. E.Z. supervised the experiments, analyzed the data, and wrote the paper.

ACKNOWLEDGMENTS

We thank Nitzan Konstantin for expert editorial assistance and all members of the E.Z. laboratory for advice and suggestions. We thank Ditsa Levanon and Elena Appel from the Yoram Groner lab, Weizmann Institute of Science, for support and advice. This study was supported by grants from the European Research Council (ERC) (#310098), the Jeanne and Joseph Nissim Foundation for Life Sciences Research, the Y. Leon Benoziyo Institute for Molecular Medicine, Beth Rom-Rymer, the Estate of David Levinson, the Jaffe Bernard and Audrey Foundation, the Georges Lustgarten Cancer Research Fund, the Estate of Bernard Bishin, and the David and Fela Shapell family. Y.A. is a consultant to the commercial company Premia Spine.

Received: June 10, 2017
Revised: July 12, 2017
Accepted: July 20, 2017
Published: August 22, 2017

REFERENCES

Abou-Khalil, R., Yang, F., Lieu, S., Julien, A., Perry, J., Pereira, C., Relaix, F., Miclau, T., Marcucio, R., and Colnot, C. (2015). Role of muscle stem cells during skeletal regeneration. Stem Cells *33*, 1501–1511.

Ai-Aql, Z.S., Alagl, A.S., Graves, D.T., Gerstenfeld, L.C., and Einhorn, T.A. (2008). Molecular mechanisms controlling bone formation during fracture healing and distraction osteogenesis. J. Dent. Res. *87*, 107–118.

Aro, H.T., and Chao, E.Y. (1993). Bone-healing patterns affected by loading, fracture fragment stability, fracture type, and fracture site compression. Clin. Orthop. Relat. Res. (*293*), 8–17.

Bach-Gansmo, F.L., Wittig, N.K., Brüel, A., Thomsen, J.S., and Birkedal, H. (2016). Immobilization and long-term recovery results in large changes in bone structure and strength but no corresponding alterations of osteocyte lacunar properties. Bone *91*, 139–147.

Bauer, O., Sharir, A., Kimura, A., Hantisteanu, S., Takeda, S., and Groner, Y. (2015). Loss of osteoblast Runx3 produces severe congenital osteopenia. Mol. Cell. Biol. *35*, 1097–1109.

Blecher, R., Krief, S., Galili, T., Biton, I., Stern, T., Assaraf, E., Levanon, D., Appel, E., Anekstein, Y., Agar, G., Groner, Y., and Zelzer, E. (2017). The proprioceptive system masterminds spinal alignment: insight into the mechanism of scoliosis. Dev. Cell *42*, 388–399.

Blitz, E., Viukov, S., Sharir, A., Shwartz, Y., Galloway, J.L., Pryce, B.A., Johnson, R.L., Tabin, C.J., Schweitzer, R., and Zelzer, E. (2009). Bone ridge patterning during musculoskeletal assembly is mediated through SCX regulation of Bmp4 at the tendon-skeleton junction. Dev. Cell *17*, 861–873.

Bolander, M.E. (1992). Regulation of fracture repair by growth factors. Proc. Soc. Exp. Biol. Med. *200*, 165–170.

Brault, V., Moore, R., Kutsch, S., Ishibashi, M., Rowitch, D.H., McMahon, A.P., Sommer, L., Boussadia, O., and Kemler, R. (2001). Inactivation of the beta-catenin gene by Wnt1-Cre-mediated deletion results in dramatic brain malformation and failure of craniofacial development. Development *128*, 1253–1264.

Brighton, C. (1984). Principals of fracture healing: Part I. The biology of fracture repair. In Instructional Course Lectures XXXIII, M. Ja, ed. (Saint Louis, MO: CV Mosby), pp. 60–82.

Carpenter, R.D., and Carter, D.R. (2010). Computational simulation of spontaneous bone straightening in growing children. Biomech. Model. Mechanobiol. *9*, 317–328.

Carter, D.R., and Orr, T.E. (1992). Skeletal development and bone functional adaptation. J. Bone Miner. Res. *7* (*Suppl 2*), S389–S395.

Chen, H.H., Tourtellotte, W.G., and Frank, E. (2002). Muscle spindle-derived neurotrophin 3 regulates synaptic connectivity between muscle sensory and motor neurons. J. Neurosci. *22*, 3512–3519.

Chen, H.H., Hippenmeyer, S., Arber, S., and Frank, E. (2003). Development of the monosynaptic stretch reflex circuit. Curr. Opin. Neurobiol. *13*, 96–102.

Cho, T.J., Gerstenfeld, L.C., and Einhorn, T.A. (2002). Differential temporal expression of members of the transforming growth factor beta superfamily during murine fracture healing. J. Bone Miner. Res. *17*, 513–520.

Christian Krettek, T.G. (2006). Principles of Internal Fixation. In Rockwood and Green's Fractures in Adults, R.W. Bucholz, C.M. Court-Brown, J.D. Heckman, and P. Tornetta, III, eds. (Lippincott Williams & Wilkins), pp. 209–257.

Connolly, J.F. (2006). Nonoperative Fracture Treatment. In Rockwood and Green's Fractures in Adults, R.W. Bucholz, C.M. Court-Brown, J.D. Heckman, and P. Tornetta, III, eds. (Lippincott Williams & Wilkins), pp. 145–208.

Currey, J.D. (2002). Bones: Structure and Mechanics (Princton University Press).

Currey, J.D. (2003). The many adaptations of bone. J. Biomech. *36*, 1487–1495.

Danielian, P.S., Muccino, D., Rowitch, D.H., Michael, S.K., and McMahon, A.P. (1998). Modification of gene activity in mouse embryos in utero by a tamoxifen-inducible form of Cre recombinase. Curr. Biol. *8*, 1323–1326.

Burr, D.B., and Allen, M.R. (2014). Basic and Applied Bone Biology (Elsevier).

Davidson, R.M., Tatakis, D.W., and Auerbach, A.L. (1990). Multiple forms of mechanosensitive ion channels in osteoblast-like cells. Pflugers Arch. *416*, 646–651.

Dykes, I.M., Tempest, L., Lee, S.I., and Turner, E.E. (2011). Brn3a and Islet1 act epistatically to regulate the gene expression program of sensory differentiation. J. Neurosci. *31*, 9789–9799.

Einhorn, T.A. (1998). The cell and molecular biology of fracture healing. Clin. Orthop. Relat. Res. (*355, Suppl*), S7–S21.

Ellman, R., Spatz, J., Cloutier, A., Palme, R., Christiansen, B.A., and Bouxsein, M.L. (2013). Partial reductions in mechanical loading yield proportional changes in bone density, bone architecture, and muscle mass. J. Bone Miner. Res. *28*, 875–885.

Ellsasser, J.C., Moyer, C.F., Lesker, P.A., and Simmons, D.J. (1975). Improved healing of experimental long bone fractures in rabbits by delayed internal fixation. J. Trauma *15*, 869–876.

Fogel, G.R., and Morrey, B.F. (1987). Delayed open reduction and fixation of ankle fractures. Clin. Orthop. Relat. Res. (*215*), 187–195.

Frost, H.M. (2001). From Wolff's law to the Utah paradigm: insights about bone physiology and its clinical applications. Anat. Rec. *262*, 398–419.

Gerstenfeld, L.C., Cullinane, D.M., Barnes, G.L., Graves, D.T., and Einhorn, T.A. (2003). Fracture healing as a post-natal developmental process: molecular, spatial, and temporal aspects of its regulation. J. Cell. Biochem. *88*, 873–884.

Glejzer, A., Laudet, E., Leprince, P., Hennuy, B., Poulet, C., Shakhova, O., Sommer, L., Rogister, B., and Wislet-Gendebien, S. (2011). Wnt1 and BMP2: two factors recruiting multipotent neural crest progenitors isolated from adult bone marrow. Cell. Mol. Life Sci. *68*, 2101–2114.

Goble, D.J., Lewis, C.A., Hurvitz, E.A., and Brown, S.H. (2005). Development of upper limb proprioceptive accuracy in children and adolescents. Hum. Mov. Sci. *24*, 155–170.

Gould, L., Abadir, P., Brem, H., Carter, M., Conner-Kerr, T., Davidson, J., DiPietro, L., Falanga, V., Fife, C., Gardner, S., et al. (2015). Chronic wound repair and healing in older adults: current status and future research. J. Am. Geriatr. Soc. *63*, 427–438.

Granit, R. (1975). The functional role of the muscle spindles–facts and hypotheses. Brain *98*, 531–556.

Huiskes, R., Ruimerman, R., van Lenthe, G.H., and Janssen, J.D. (2000). Effects of mechanical forces on maintenance and adaptation of form in trabecular bone. Nature *405*, 704–706.

Husain, S.N., King, E.C., Young, J.L., and Sarwark, J.F. (2008). Remodeling of birth fractures of the humeral diaphysis. J. Pediatr. Orthop. *28*, 10–13.

Ilizarov, G.A. (1990). Clinical application of the tension-stress effect for limb lengthening. Clin. Orthop. Relat. Res. (*250*), 8–26.

Inoue, K., Ozaki, S., Ito, K., Iseda, T., Kawaguchi, S., Ogawa, M., Bae, S.C., Yamashita, N., Itohara, S., Kudo, N., and Ito, Y. (2003). Runx3 is essential for the target-specific axon pathfinding of trkc-expressing dorsal root ganglion neurons. Blood Cells Mol. Dis. *30*, 157–160.

Isern, J., García-García, A., Martín, A.M., Arranz, L., Martín-Pérez, D., Torroja, C., Sánchez-Cabo, F., and Méndez-Ferrer, S. (2014). The neural crest is a source of mesenchymal stem cells with specialized hematopoietic stem cell niche function. eLife *3*, e03696.

Kahn, J., Shwartz, Y., Blitz, E., Krief, S., Sharir, A., Breitel, D.A., Rattenbach, R., Relaix, F., Maire, P., Rountree, R.B., et al. (2009). Muscle contraction is necessary to maintain joint progenitor cell fate. Dev. Cell *16*, 734–743.

Kettelkamp, D.B., Hillberry, B.M., Murrish, D.E., and Heck, D.A. (1988). Degenerative arthritis of the knee secondary to fracture malunion. Clin. Orthop. Relat. Res. (*234*), 159–169.

Kokkorogiannis, T. (2004). Somatic and intramuscular distribution of muscle spindles and their relation to muscular angiotypes. J. Theor. Biol. *229*, 263–280.

Lam, S.J. (1964). The place of delayed internal fixation in the treatment of fractures of the long bones. J. Bone Joint Surg. Br. *46*, 393–397.

Lee, H.S., Millward-Sadler, S.J., Wright, M.O., Nuki, G., and Salter, D.M. (2000). Integrin and mechanosensitive ion channel-dependent tyrosine phosphorylation of focal adhesion proteins and beta-catenin in human articular chondrocytes after mechanical stimulation. J. Bone Miner. Res. *15*, 1501–1509.

Levanon, D., and Groner, Y. (2009). Runx3-deficient mouse strains circa 2008: resemblance and dissimilarity. Blood Cells Mol. Dis. *43*, 1–5.

Levanon, D., Bettoun, D., Harris-Cerruti, C., Woolf, E., Negreanu, V., Eilam, R., Bernstein, Y., Goldenberg, D., Xiao, C., Fliegauf, M., et al. (2002). The Runx3 transcription factor regulates development and survival of TrkC dorsal root ganglia neurons. EMBO J. *21*, 3454–3463.

Lian, J.B., Javed, A., Zaidi, S.K., Lengner, C., Montecino, M., van Wijnen, A.J., Stein, J.L., and Stein, G.S. (2004). Regulatory controls for osteoblast growth and differentiation: role of Runx/Cbfa/AML factors. Crit. Rev. Eukaryot. Gene Expr. *14*, 1–41.

Logan, M., Martin, J.F., Nagy, A., Lobe, C., Olson, E.N., and Tabin, C.J. (2002). Expression of Cre Recombinase in the developing mouse limb bud driven by a Prxl enhancer. Genesis *33*, 77–80.

Maeda, N., Osawa, K., Masuda, T., Hakeda, Y., and Kumegawa, M. (1985). Postnatal development of the anulospiral endings of Ia fibers in muscle spindles of mice. Acta Anat. (Basel) *124*, 42–46.

Maier, A. (1997). Development and regeneration of muscle spindles in mammals and birds. Int. J. Dev. Biol. *41*, 1–17.

Moore, J.C. (1984). The Golgi tendon organ: a review and update. Am. J. Occup. Ther. *38*, 227–236.

O'Donovan, K.J., Ma, K., Guo, H., Wang, C., Sun, F., Han, S.B., Kim, H., Wong, J.K., Charron, J., Zou, H., et al. (2014). B-RAF kinase drives developmental axon growth and promotes axon regeneration in the injured mature CNS. J. Exp. Med. *211*, 801–814.

Oliveira Fernandes, M., and Tourtellotte, W.G. (2015). Egr3-dependent muscle spindle stretch receptor intrafusal muscle fiber differentiation and fusimotor innervation homeostasis. J. Neurosci. *35*, 5566–5578.

Osawa, K., Maeda, N., Sato, M., Kawasaki, T., Masuda, T., Yamamoto, Y., Hakeda, Y., Ukai, M., Watanabe, Y., Suwa, T., et al. (1988). Postnatal development of the annulospiral endings of Ia fibers in muscle spindles of the mouse temporal muscle. Anat. Anz. *167*, 253–257.

Pickett, K., and Konczak, J. (2009). Measuring kinaesthetic sensitivity in typically developing children. Dev. Med. Child Neurol. *51*, 711–716.

Probst, A., and Spiegel, H.U. (1997). Cellular mechanisms of bone repair. J. Invest. Surg. *10*, 77–86.

R Development Core Team (2016). R: A language and environment for statistical computing (Vienna, Austria, R Foundation for Statistical Computing).

Ring, D. (2005). Treatment of the neglected distal radius fracture. Clin. Orthop. Relat. Res. (*431*), 85–92.

Robling, A.G., Castillo, A.B., and Turner, C.H. (2006). Biomechanical and molecular regulation of bone remodeling. Annu. Rev. Biomed. Eng. *8*, 455–498.

Rot, C., Stern, T., Blecher, R., Friesem, B., and Zelzer, E. (2014). A mechanical Jack-like mechanism drives spontaneous fracture healing in neonatal mice. Dev. Cell *31*, 159–170.

Sanz, N., Díez-Fernández, C., Alvarez, A.M., Fernández-Simón, L., and Cascales, M. (1999). Age-related changes on parameters of experimentally-induced liver injury and regeneration. Toxicol. Appl. Pharmacol. *154*, 40–49.

Schindeler, A., McDonald, M.M., Bokko, P., and Little, D.G. (2008). Bone remodeling during fracture repair: The cellular picture. Semin. Cell Dev. Biol. *19*, 459–466.

Schultz, A.H. (1939). Notes on diseases and healed fractures of wild apes and their bearing on the antiquity of pathological conditions in man. Bull. Hist. Med. *7*, 571–582.

Schultz, A.H. (1944). Age changes and variability in gibbons. A morphological study on a population sample of a man-like ape. Am. J. Phys. Anthropol. *2*, 1–129.

Shapiro, F. (2008). Bone development and its relation to fracture repair. The role of mesenchymal osteoblasts and surface osteoblasts. Eur. Cell. Mater. *15*, 53–76.

Sharir, A., Stern, T., Rot, C., Shahar, R., and Zelzer, E. (2011). Muscle force regulates bone shaping for optimal load-bearing capacity during embryogenesis. Development *138*, 3247–3259.

Shwartz, Y., Farkas, Z., Stern, T., Aszódi, A., and Zelzer, E. (2012). Muscle contraction controls skeletal morphogenesis through regulation of chondrocyte convergent extension. Dev. Biol. *370*, 154–163.

Soukup, T. (1983). The number, distribution and size of Golgi tendon organs in developing and adult rat muscles. Physiol. Bohemoslov. *32*, 211–224.

Stricker, S., Fundele, R., Vortkamp, A., and Mundlos, S. (2002). Role of Runx genes in chondrocyte differentiation. Dev. Biol. *245*, 95–108.

Takeoka, A., Vollenweider, I., Courtine, G., and Arber, S. (2014). Muscle spindle feedback directs locomotor recovery and circuit reorganization after spinal cord injury. Cell *159*, 1626–1639.

Thompson, Z., Miclau, T., Hu, D., and Helms, J.A. (2002). A model for intramembranous ossification during fracture healing. J. Orthop. Res. *20*, 1091–1098.

Tourtellotte, W.G., and Milbrandt, J. (1998). Sensory ataxia and muscle spindle agenesis in mice lacking the transcription factor Egr3. Nat. Genet. *20*, 87–91.

Vortkamp, A., Pathi, S., Peretti, G.M., Caruso, E.M., Zaleske, D.J., and Tabin, C.J. (1998). Recapitulation of signals regulating embryonic bone formation during postnatal growth and in fracture repair. Mech. Dev. *71*, 65–76.

Wann, A.K., Zuo, N., Haycraft, C.J., Jensen, C.G., Poole, C.A., McGlashan, S.R., and Knight, M.M. (2012). Primary cilia mediate mechanotransduction through control of ATP-induced Ca2+ signaling in compressed chondrocytes. FASEB J. *26*, 1663–1671.

Weiner, S., and Wagner, H.D. (1998). The material bone: structure-mechanical function relations. Annu. Rev. Mat. Sci *28*, 271–298.

Wilkins, K.E. (2005). Principles of fracture remodeling in children. Injury *36* (*Suppl 1*), A3–A11.

Windhorst, U. (2007). Muscle proprioceptive feedback and spinal networks. Brain Res. Bull. *73*, 155–202.

Yoshida, C.A., Yamamoto, H., Fujita, T., Furuichi, T., Ito, K., Inoue, K., Yamana, K., Zanma, A., Takada, K., Ito, Y., and Komori, T. (2004). Runx2 and Runx3 are essential for chondrocyte maturation, and Runx2 regulates limb growth through induction of Indian hedgehog. Genes Dev. *18*, 952–963.

Yun, M.H. (2015). Changes in regenerative capacity through lifespan. Int. J. Mol. Sci. *16*, 25392–25432.

Zelzer, E., Blitz, E., Killian, M.L., and Thomopoulos, S. (2014). Tendon-to-bone attachment: from development to maturity. Birth Defects Res. C Embryo Today *102*, 101–112.

Cell Reports, Volume *20*

Supplemental Information

The Proprioceptive System Regulates Morphologic Restoration of Fractured Bones

Ronen Blecher, Sharon Krief, Tal Galili, Eran Assaraf, Tomer Stern, Yoram Anekstein, Gabriel Agar, and Elazar Zelzer

CellPress

Supplemental Experimental Procedures

RESOURCE TABLE

REAGENT or RESOURCE	SOURCE	IDENTIFIER
Chemicals, Peptides, and Recombinant Proteins		
Clorketam (ketamine)	Vetoquinol, France	N/A
Xylazine (Sedaxylan)	Eurovet, Netherlands	23076-35-9
Buprenorphine	Vetmarket, Israel	163451
Isoflurane	AbbVie, UK	B506
Botulinum toxin A (Botox)	Allergan	N/A
Phosphate-buffered saline (PBS)	Gibco by Life Technologies, UK	14200-067
Experimental Models: Organisms/Strains		
Mouse: ICR	Envigo, Israel	Hsd:ICR (CD-1)
Mouse: C57BL/6	Envigo, Israel	C57BL/6JOlaHsd
Software and Algorithms		
R	R Development Core Team, 2016	https://www.r-project.org/
